# Fine particulate (PM_2.5_) dynamics during rapid urbanization in Beijing, 1973–2013

**DOI:** 10.1038/srep23604

**Published:** 2016-03-31

**Authors:** Lijian Han, Weiqi Zhou, Weifeng Li

**Affiliations:** 1 State Key Laboratory of Urban and Regional Ecology, Research Center for Eco-Environmental Sciences, Chinese Academy of Sciences, Beijing 100085, China

## Abstract

PM_2.5_ has been given special concern in recent years when the air quality monitoring station started recording. However, long-term PM_2.5_ concentration dynamic analysis cannot be taken with the limited observations. We therefore estimated the PM_2.5_ concentration using meteorological visibility data in Beijing. We found that 71 ± 17% of PM_10_ were PM_2.5_, which contributed to visibility impairment (y = 332.26e^−0.232x^; R^2^ = 0.75, P < 0.05). We then reconstructed a time series of annual PM_2.5_ from 1973 to 2013, and examined its relationship with urbanization by indicators of population, gross domestic production (GDP), energy consumption, and number of vehicles. Concluded that 1) Meteorological conditions were not the major cause of PM_2.5_ increase from 1973 to 2013; 2) With population and GDP growth, PM_2.5_ increased significantly (R^2^ = 0.5917, P < 0.05; R^2^ = 0.5426, P < 0.05); 3) Intensive human activity could change air quality in a short period, as observed changes in the correlations of PM_2.5_ concentration with energy consumption and number of vehicles before and after 2004, respectively. The success of this research provides an easy way in reconstructing long-term PM_2.5_ concentration with limited PM_2.5_ observation and meteorological visibility, and insight the impact of urbanization on air quality.

Due to rapid urbanization in the last century, more than half the world’s human population now live in cities^1^. Human activities, especially in large cities, have led to an improvement in material wealth and a higher standard of living, but have also caused severe environmental problems such as air pollution. This is particularly true in the rapidly developing mega cities of developing countries^2^,^3^.

Fine particulate matter is a major air pollutant, which causes visibility degradation and is a toxic component that threatens public health in many large cities^4^,^5^,^6^. Generally, PM_2.5_ concentrations can be monitored with an air quality monitoring network, remote sensing images, and meteorological visibility records^7^. Air quality networks have long been established in developed countries, and in recent years have been established in a limited number of large cities in developing countries where rapid urbanization has negatively impacted urban air quality^2^. Remote sensing has been paid special concern on PM_2.5_ retrieval; however, it still needs further algorithmic approaches to improve its retrieval accuracy, and remains limited in regards to long-term series image availability^7^. Meteorological visibility data, which has been available since the 1970 s in most major cities of the world, provides another way to determine PM_2.5_ concentrations by calibrating the relationship between visibility and PM_2.5_ observation records^8^,^9^.

PM_2.5_ concentration is a typical indicator for urban air quality, and is impacted by rapid urbanization progress. The present research utilized ground measurements of PM_2.5_ concentration, meteorological visibility data, and urbanization indicators 1) to determine the correlation between visibility and PM_2.5_ concentration; and 2) to quantify PM_2.5_ concentration dynamics and its relationship with urbanization in Beijing, a typical large Chinese city.

## Results

**Figure 1:**
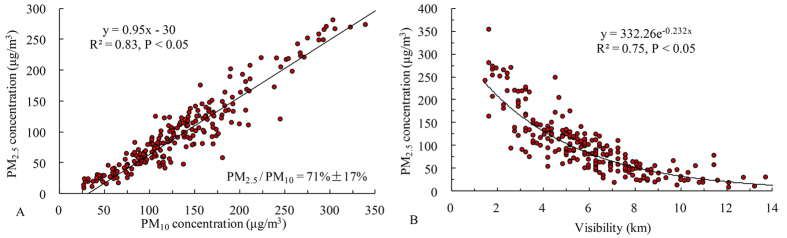
Proportion of PM_2.5_ in PM_10_ (**A**) and relationship between PM_2.5_ concentration and visibility (**B**) on stable meteorological days.

**Figure 2:**
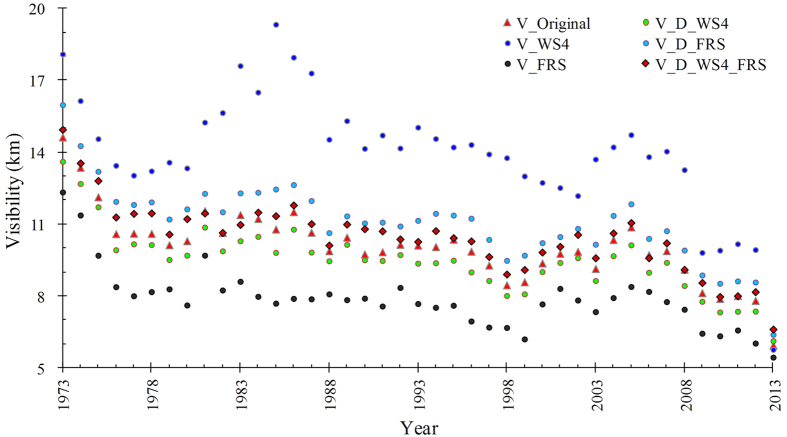
Visibility in Beijing from 1973 to 2012. V_Original is the original annual mean visibility; V_WS4 is annual mean visibility on days with average wind speeds >4 m/s; V_FRS is annual mean visibility on days with fog, rain, or snow; V_D_WS4 is annual mean visibility with wind speed (>4 m/s) days eliminated; V_D_FRS is annual mean visibility, with fog, rain, or snow days eliminated; V_D_WS4_FRS is annual mean visibility, with fog, rain, snow, or wind speed (>4 m/s) days eliminated.

Results showed that PM_2.5_ (71 ± 17%) was the major component of PM_10_ in Beijing by analyzed with 223 days under stable meteorological conditions (Fig. 1A). In addition, the increase in PM_2.5_ contributed to visibility impairment significantly (R^2^ = 0.75, P < 0.05; Fig. 1B). Annual mean visibility decreased in Beijing from 1973 to 2013 (Fig. 2). Moreover, annual mean visibility on days with only wind speeds greater than 4 m/s (V_WS4) were greater than other conditions, indicating strong wind is the major force to remove the air pollutants.

**Figure 3:**
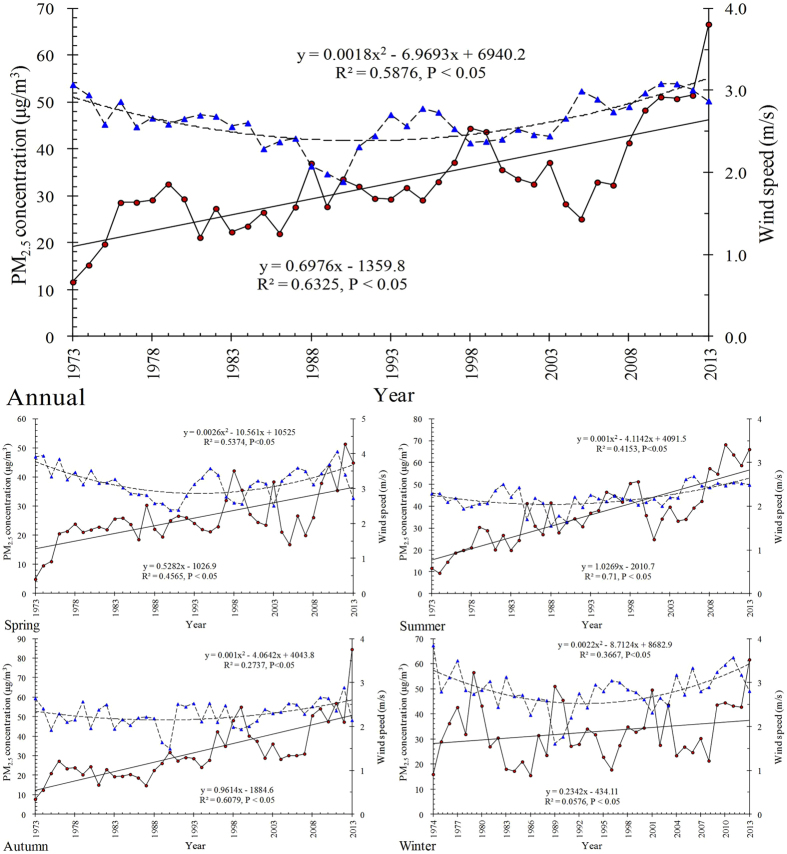
Annual and seasonal mean PM_2.5_ concentrations and wind speeds in Beijing from 1973 to 2013 on stable meteorological days. Dark red and blue dots represent annual PM_2.5_ concentration and wind speed, respectively. Winter includes January, February, and the previous December; Spring includes March, April, and May; Summer includes June, July, and August; and Autumn includes September, October, and November.

The annual mean PM_2.5_ concentration under stable meteorological condition increased significantly (R^2^ = 0.6325, P < 0.05; Fig. 3), with wind speed showed a “U-shape” trend which is relative stable, thus, indicated human activities would be the major reason that result in the increase of PM_2.5_ concentration(Fig. 3). The seasonal mean increase of PM_2.5_ concentration was increased stronger in summer (slope = 1.0269) and autumn (slope = 0.9614) than that in spring (slope = 0.5282) and winter (slope = 0.2342). Moreover, PM_2.5_ concentration increased largest in summer, but no significant trend was observed in winter during 1973–2013.

**Figure 4:**
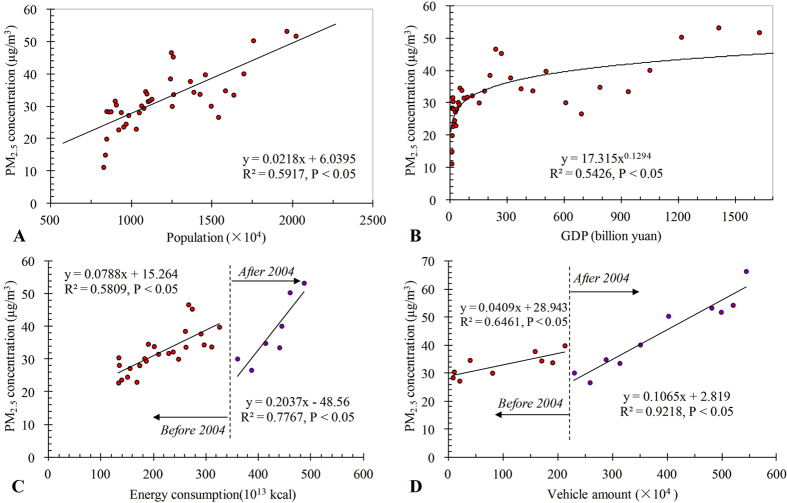
Correlation between urbanization indicators (Population (**A**) from 1973 to 2011, GDP (**B**) from 1973 to 2011, energy consumption (**C**) from 1980 to 2010, and number of vehicles (**D**) from 1978 to 2013) and PM_2.5_ concentration on stable meteorological days in Beijing.

Urbanization indicators were significantly correlated with PM_2.5_ concentration at Beijing. Both population (R^2^ = 0.5917, P < 0.05; Fig. 4A) and GDP (R^2^ = 0.5426, P < 0.05; Fig. 4B) were positively correlated with PM_2.5_ concentration during 1973–2013, indicating the increasing human activities is highly attribute to the increase of PM_2.5_ concentration. Energy consumption also could contribute to the increase the PM_2.5_ concentration (Fig. 4C). The slopes between PM_2.5_ concentration and energy consumption were changed after 2004. While, similar correlation was also obtained between PM_2.5_ concentration and vehicle amount before and after 2004 (Fig. 4D).

## Discussion

PM_2.5_ is an important component in PM_10_. However, the ratio of PM_2.5_ to PM_10_ varies among different areas, for example, 33% in Jeddah City, Saudi Arabia, and between 45–60% in Greece^10^,^11^,^12^. PM_2.5_ can easily enter the human respiratory system and cause serious health impacts, while larger particles are not able to penetrate as deeply and therefore cause less serious health impacts^6^. Thus, at the same particulate pollution levels, higher ratios of PM_2.5_ to PM_10_ indicate the potential for greater negative impacts on human health. In the present study, the ratio in Beijing was found to be 71% ± 17%, indicating the probability of significant impact on health. Furthermore, both PM_10_ and PM_2.5_ are the major course of visibility impairment. If PM_2.5_ is not the major component in PM_10_, our method cannot be applied, thus the accuracy of long-term PM_2.5_ concentration is highly correlated with the consistency of the correlation between PM_2.5_ concentration and visibility during the study period. The particulate data collected in this research was only available for a year, and further calibration of the ratio and the relationship between PM_2.5_ concentration and visibility at longer time scale is strongly suggested to improve the accuracy in determining long-term PM_2.5_ dynamics at different cities.

The negative impacts of urbanization on the environment, especially on air, have been given special attention in recent years. For instance, the Environmental Kuznets Curve (EKC) found an inverse U-type relationship between the urban eco-environment and the economy, with the turning point of the U-curve normally at a per capita income of $8000. However, we did not observe an inverse U-type relationship between the economy and PM_2.5_ concentration, indicating that Beijing may not have reached the turning point in the EKC U-type curve. The relationship between energy consumption, the number of vehicles, and PM_2.5_ concentration (Fig. 4C,D) also indicated that the economy was not the only influence on the air environment. Different relationships were observed before and after 2004, for example, indicating the strong impact of human activity on environmental improvement.

Urban systems are not naturally developed, but are always influenced by human activities^1^. Intense human activity can change the urban environment over a short period. This was also observed in this work as the relationship between PM_2.5_ and urbanization indicators showed. At beginning, Beijing’s development was highly depended on heavy industries that made the GDP increase while polluted the atmospheric environment, however, the policy was changed thanks to the Olympic Games and its related environmental protection activity^13^. After Beijing was selected as the host city of the 2008 Olympic Games, several environmental protection policies were established, including the relocation of heavy industry to outside of Beijing. These activities, which took great effect from 2004, contributed to the reduction in the concentration of PM_2.5_. After the improvement in air quality in 2004, however, the rapid increase in the number of vehicles provided a new source of PM_2.5_, with the significant relationship observed indicating the strong negative impact of vehicle emissions on urban air quality after 2004 (R^2^ = 0.9218, P < 0.05; Fig. 4D). Thus, the relationship between PM_2.5_ concentration and urbanization indicators showed increase, decrease, and increase again from 1973 to 2013.

Similar to other mega cities in China, Beijing will continue its rapid urbanization for another decade as part of the National New-type Urbanization Plan stratagem (2014 to 2020) designed by the Chinese Central Government. From now until 2020, the national urbanization rate is planned to reach around 60% on the basis of the 52.6% achieved in 2012. Such rapid increase will bring more intensive social and economic activities, which will directly affect the urban environment. Thus, the development of better strategies for the control and reduction of air pollution without compromising economic growth is essential for China’s continued urbanization.

## Materials and Methods

### Daily visibility and meteorological data

Daily visibility, wind speed at 10 m height, and indicators for occurrences of fog, rain, and snow were obtained from Global Summary of the Day from the National Climate Data Center of the U.S. Department of Commerce. These data have been recorded in Beijing since 1973, allowing long-term series analysis of visibility in order to illustrate particulate pollution dynamics in the city.

### Social-economic data

Data on the annual urban population, gross domestic production (GDP), energy consumption, and numbers of vehicles in Beijing were collected from the Beijing 60 Yearbook, and were further correlated with the annual PM_2.5_ dynamics to understand the impact of urbanization on urban air quality in a typical Chinese megacity.

### Daily PM_2.5_ and PM_10_ data

Daily records of PM_2.5_ and PM_10_ concentrations in Beijing were obtained from the China National Environmental Monitoring Centre from October 2013 to September 2014, covering an entire year with both high and low pollution days and various meteorological conditions following the ways that set under the Specifications and Test Procedures for PM_10_ and PM_2.5_ Sampler (HJ-93-2013) by Ministry of Environmental Protection of China (available at: http://www.mep.gov.cn/).

### Visibility under stable meteorological condition

Visibility under stable meteorological condition could illustrate the local particulate pollution condition, we therefore eliminate the visibility under instable meteorological conditions: (1) visibility under rain, fog, and snow days was firstly eliminated to minimize visibility impairment from natural precipitation; (2) and then, visibility with wind speed faster than 4 m/s, which was deduced in our previous research when comparing wind speed with air quality index (AQI)^4^, was also eliminated to ease the wind speed’s positive impact on air quality improvement via carry and spread the pollutant to the downward area.

### Estimation of annual PM_2.5_ concentration from visibility

The relationship between PM_2.5_ and PM_10_ was firstly examined to ensure that PM_2.5_ was the major component in PM_10_ that caused the visibility impairment. The correlation between daily PM_2.5_ concentration and visibility was then obtained under stable meteorological conditions. From this, 40 years of PM_2.5_ concentration dynamics were finally estimated.

### Correlation analysis

Annual and seasonal stable PM_2.5_ concentrations were firstly correlated with annual and seasonal stable wind speeds during the 40 years to understand stable meteorological conditions has less impact on local emitted PM_2.5_ dynamics. Correlations between PM_2.5_ and population, GDP, energy consumption, and number of vehicles were then examined to understand the impact of urbanization on PM_2.5_ concentrations in the typical Chinese megacity, Beijing.

## Additional Information

**How to cite this article**: Han, L. *et al*. Fine particulate (PM_2.5_) dynamics during rapid urbanization in Beijing, 1973–2013. *Sci. Rep.* **6**, 23604; doi: 10.1038/srep23604 (2016).
